# Architected Spatially Deterministic Multiscale Phase Segregation in Block Copolymer Composites for Augmented Radiative Cooling

**DOI:** 10.1002/advs.77913

**Published:** 2026-09-27

**Authors:** Mingxin Feng, Feiyang Zhou, Haoran Cai, Wenchao Zhai, Shuangjiang Feng, Yanmei Liu, Xiaohai Bu, Man He, Dudong Feng, Shuang Liang, Yuming Zhou

**Affiliations:** ^1^ School of Chemistry and Chemical Engineering, Jiangsu Optoelectronic Functional Materials and Engineering Research Center, Jiangsu Key Laboratory of Advanced Optical Functional Film Materials and Technologies Southeast University Nanjing China; ^2^ School of Energy and Environment Southeast University Nanjing China; ^3^ State Key Laboratory of Analytical Chemistry for Life Science, School of Chemistry and Chemical Engineering Nanjing University Nanjing China; ^4^ School of Materials Science and Engineering, Jiangsu Key Laboratory of Advanced Structural Materials and Application Technology Nanjing Institute of Technology Nanjing China

**Keywords:** block copolymer composite, macroscopic phase segregation, microphase separation, passive radiative cooling, thermal dissipation

## Abstract

Passive radiative cooling, which reflects solar irradiation while emitting heat through the atmospheric window, provides a sustainable solution for zero‐energy thermal management. Simultaneously achieving high solar reflectivity and infrared emissivity remains challenging since the structural parameters governing light scattering and thermal radiation are intrinsically coupled. A block copolymer composite (denoted SEDO) of polystyrene‐*block*‐poly(ethylene‐*ran*‐butylene)‐*block*‐polystyrene (SEBS) with 9,10‐dihydro‐9‐oxa‐10‐phosphaphenanthrene‐10‐oxide (DOPO) was judiciously designed and architected through multiscale phase segregation to enable decoupled structure‐determined spectral regulation. Macroscopic phase segregation in SEDO endows three‐dimensionally interconnected porous skeleton that enhances broadband Mie scattering, resulting in a high solar reflectivity of 97.2%. Meanwhile, DOPO fillers that selectively located in the polystyrene domains introduce strong molecular vibrational absorption within the atmospheric window. The cylindrical microphase separation morphology enables an emissivity of 95.5% with only 5 vol% additive. Benefiting from this decoupled optical regulation, the composite achieves sub‐ambient cooling of ∼8.1°C under clear‐sky conditions and ∼6.3°C under cloudy conditions, while maintaining efficient heat dissipation under high heat flux. Combined with excellent mechanical flexibility, flame retardancy, and environmental stability, this multiscale phase segregation strategy provides a versatile platform for augmented radiative cooling in practical thermal‐management applications via crafting spatially deterministic block copolymer composites.

## Graphical abstract

Multiscale phase segregation in block copolymers enables spatially deterministic structuring from molecular to macroscopic scales, decoupling solar scattering and infrared emission for augmented radiative cooling.

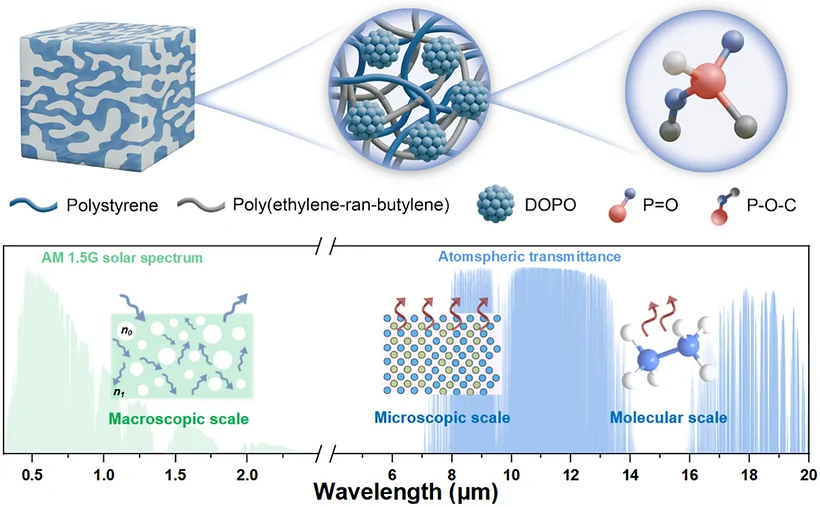

## 1 Introduction

As integration density and component miniaturization continue to escalate, heat generation and accumulation within modern electronics becomes increasingly localized and intensified, giving rise to microscale hotspots that surpass the heat dissipation capacity of conventional cooling methods [1, 2, 3, 4]. Elevated temperatures inevitably lead to pronounced performance degradation, diminished reliability, and reduced operational lifetimes. Hence, thermal management technologies that simultaneously offer effective heat dissipation, lightweight, minimal structural complexity, ease of electronic integration, and mechanical flexibility are not merely advantageous but critically essential for sustaining the continued scaling and performance evolution of next‐generation electronics. Radiative cooling allows heat dissipation without energy consumption by passively emitting infrared radiation to outer space through the atmospheric transparency window (8–13 µm) [5, 6, 7]. When combined with strong solar reflection (0.3–2.5 µm), this mechanism can achieve sub‑ambient cooling even under direct sunlight. Moreover, polymer‐based radiative cooling materials are particularly attractive due to their intrinsic lightweight nature, mechanical flexibility, and compatibility with scalable fabrication methods (e.g., solution processing and roll‐to‐roll manufacturing), enabling their implementation as thin films or flexible sheets for seamless integration into electronic devices. Consequently, polymer‐based radiative cooling materials represent a promising solution to localized overheating in high‑density and high‑power electronics [8, 9].

**FIGURE 1:**
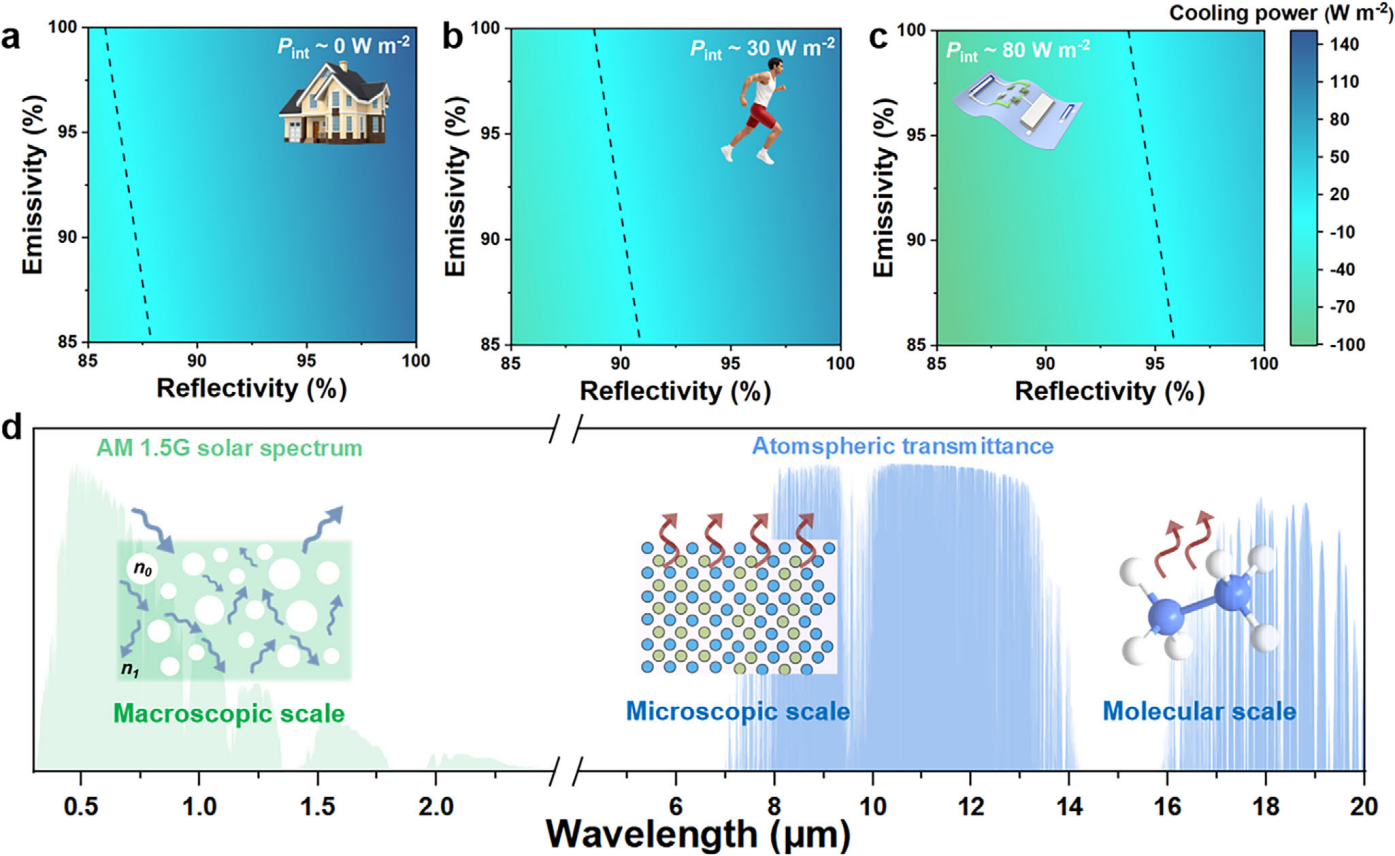
Correlations between optical properties and multiscale structural design for effective radiative cooling. (a‐c) Effects of solar reflectivity and infrared emissivity on the net cooling power under different external heat input powers. (d) Influence of structural design from the molecular to molecular to microscopic and macroscopic scales on solar reflectivity and infrared emissivity.

In polymer‐based radiative cooling materials, the synergistic optimization of high solar reflectivity and high infrared emissivity is fundamentally governed by the rational design of structural parameters. Depending on the heat flux density, radiative cooling efficiency can be tailored by modulating the material's molecular vibration, microstructure and macroscopic phase segregation to match distinctive cooling demands. For instance, at low heat fluxes (e.g., 0–30 W m^−2^ in building envelopes), conventional radiative organic coatings or polymeric films suffice would afford efficient heat dissipation, whereas for moderate to high fluxes (e.g., 30–80 W m^−2^ in integrated circuits or wearable electronics), the cooling layer must be integrated with thermally emissive inorganic components or microstructured interfaces to efficiently extract and dissipate heat without compromising flexibility or device integration (Figure 1a–c). However, the structural features governing solar scattering and those regulating mid‐infrared radiation are often interrelated and even competitive [10, 11]. Specifically, Mie‐scattering structures that enhance solar reflection may reduce the radiative efficiency in the mid‐infrared region, whereas the introduction of molecular vibrational features that strengthen infrared emission may simultaneously induce parasitic absorption of solar radiation. Therefore, rationally design and architect radiative cooling composite from molecular to microscopic and macroscopic scales are of critical importance to maintain high solar reflectivity and simultaneously enhance infrared emissivity (Figure 1d).

Across different structural scales, polymer‐based radiative cooling materials have been rationally engineered to regulate solar reflection and infrared emission [12, 13, 14, 15]. At the molecular level, characteristic functional groups render distinctive molecular vibrational modes that fundamentally dictate mid‐infrared absorption behaviors. Through judicious molecular engineering, near‐ideal absorption profiles across the mid‐infrared spectrum can be attained, thereby enabling tunable infrared emissivity [16, 17, 18]. The incorporation of ether moieties, fluorine‐containing groups, and phosphorus‐ or phosphine‐based functionalities, either as pendant substituents or through additive inclusion, imparted the desired infrared spectral response within the atmospheric windows, thereby promoting passive radiative cooling. Moreover, chain conformation and crystalline structure engineering of polymer fibers, achieved through high draw ratios and rapid solvent evaporation, can alter the infrared vibrational intensity of nylon 6, thereby enabling a transition between infrared emission and transmission [19]. Beyond molecular and conformational design, microstructural engineering of polymer composites, encompassing directional patterning to fabricate photonic crystals [20, 21, 22] and metasurfaces [23, 24, 25], has demonstrated great potential for infrared modulation, owing to synergistic absorption mechanisms and ordered architectures. Notably, entropy‐driven phase engineering strategy that enables the balanced coexistence of three strongly coupled crystalline phases promotes lattice vibrational absorption across the mid infrared region, and has been demonstrated to impart outstanding broadband infrared functionality to materials [26, 27]. Meanwhile, solar reflectivity is typically realized through macroscopic engineering by constructing Mie scattering structure and refractive index contrast to regulate the spectral region and intensity of reflection [28, 29]. However, despite these advances, most existing strategies primarily regulate the overall composition, morphology, or optical interfaces of materials, whereas the spatial distribution of infrared‐active components within polymer matrices remains largely determined by blending behavior, phase compatibility, or uncontrolled dispersion processes [30, 31, 32]. Therefore, introducing an additional structural dimension capable of precisely regulating the spatial localization of functional components is highly desirable. Block copolymer microphase separation provides such an opportunity. Owing to the thermodynamic incompatibility between different polymer blocks, block copolymers can spontaneously form nanoscale domains with tunable morphology, characteristic dimensions, and interfacial structures [33, 34, 35]. Unlike randomly blended composites, these self‐assembled domains provide a structural basis for regulating the spatial distribution and local concentration of functional components, thereby offering new possibilities for controlling light‐matter interactions at the nanoscale. Nevertheless, the potential of block copolymer microphase separation as a spatial regulation strategy for radiative cooling remains largely unexplored. Within this multiscale design framework, block copolymer composites provide a promising route for constructing spatially deterministic structures by integrating molecular composition, microphase‐separated domains, and macroscopic phase morphology.

Herein, we designed spatially deterministic block copolymer composites with engineered multiscale phase segregation, enabling decoupled spectral responses and structure‐dependent enhancement in radiative cooling performance. The composite (denoted SEDO) consists of a polystyrene‐*block*‐poly(ethylene‐*ran*‐butylene)‐*block*‐polystyrene (SEBS) matrix with the functional filler 9,10‐dihydro‐9‐oxa‐10‐phosphaphenanthrene‐10‐oxide (DOPO) preferentially located in the polystyrene domains. At the molecular scale, the incorporation of DOPO introduces strong vibrational absorption within the atmospheric window, thereby enhancing mid‐infrared emission. At the microscopic scale, the intrinsic microphase separation of SEBS regulates the spatial distribution of DOPO through its preferential localization within the PS domains. The formation of cylindrical microphase‐separated structures enables a high infrared emissivity of 95.5% at a DOPO loading of only 5 vol%. At the macroscopic scale, solvent evaporation‐induced phase segregation generates a 3D interconnected porous structure that enhances broadband solar scattering. The relatively high refractive index of DOPO further improves Mie scattering efficiency, allowing the composite to overcome the optical limitations of pristine SEBS and achieve an average solar reflectivity of 97.2% across the solar spectrum. Therefore, the SEDO composite rendered sub‐ambient cooling of ∼8.1°C under clear‐sky conditions and ∼6.3°C under cloudy conditions in outdoor cooling measurements. This multiscale phase segregation strategy overcomes the optical limitations of conventional single‐component materials and establishes a new paradigm for rationally incorporating distinctive functional components and constructing spatially deterministic composite structure, achieving robust and high‐performance radiative cooling under diverse external heat inputs.

## 2 Results and Discussion

### 2.1 Multiscale Phase Segregation in SEDO Composite

**FIGURE 2:**
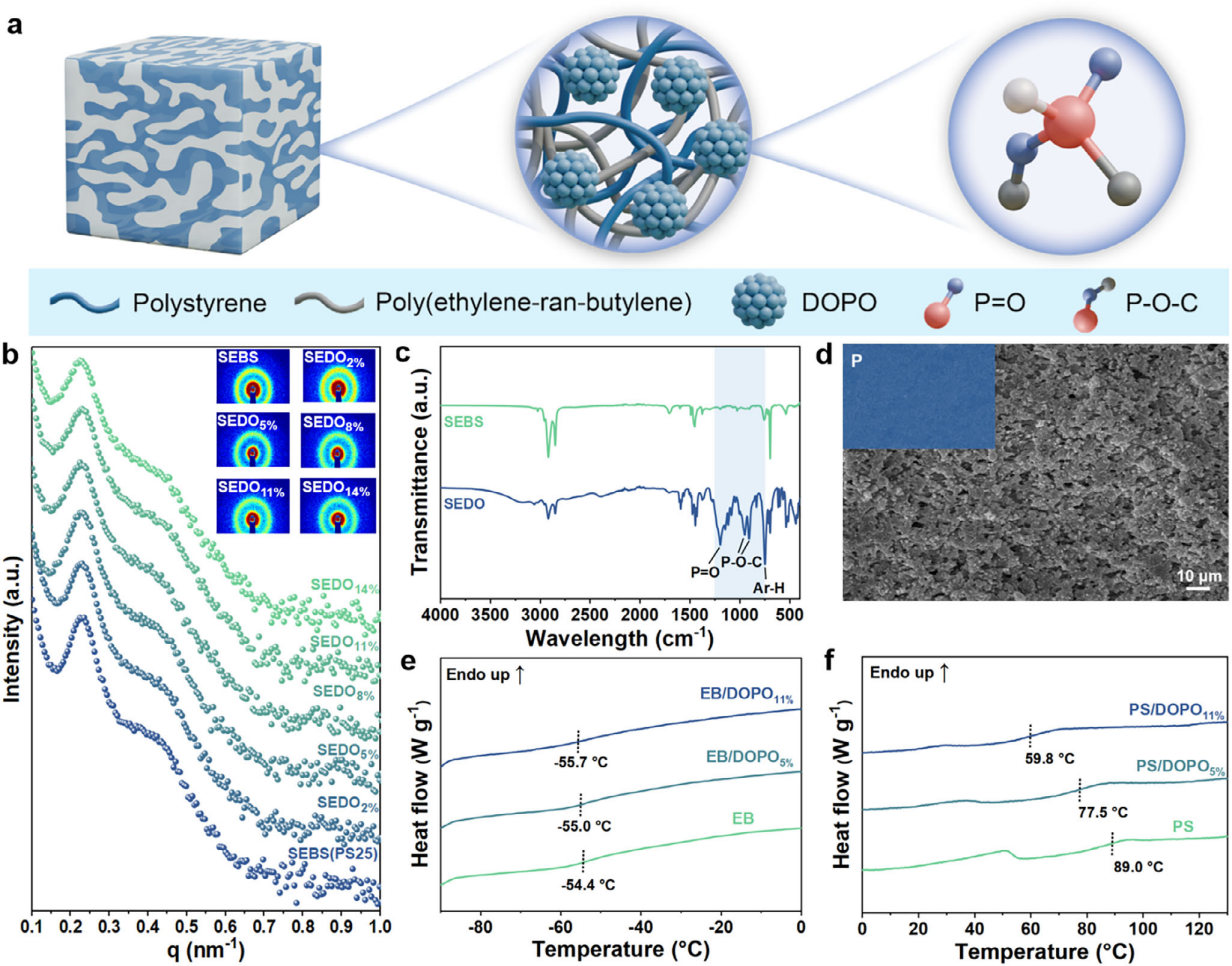
Multiscale phase segregation in SEDO. (a) Schematic of the spatially deterministic multiscale (macroscopic, microscopic, and molecular) phase segregation structure of the SEDO composite. (b) SAXS patterns of SEDO with different DOPO loadings. (c) FTIR spectra of SEBS and SEDO. (d) SEM image of SEDO; the inset shows the elemental mapping of P. (e, f) DSC curves of the EB and PS blocks at different DOPO contents.

The multiscale phase segregation of SEDO originated from the synergistic effect of solvent‐evaporation‐induced macroscopic phase separation and self‐assembly‐driven microphase separation of SEBS. The SEBS (*M*_n_ ≈ 2.15 × 10^5^ g/mol, polydispersity ≈ 1.12, polystyrene (PS) content ≈ 25 wt%, details in *Supporting Information*) and DOPO of different loading ratios were dissolved in chloroform, followed by mixing with isopropanol and casting into a polytetrafluoroethylene mold before solvent evaporation. During solvent evaporation, the quick removal of chloroform yielded unfavored interaction between isopropanol and SEBS, creating macroscopic phase segregation and formation of a micron‐scale porous framework after the evaporation of isopropanol [36]. Meanwhile, spontaneous microphase separation yielded nanoscopic domains due to the intrinsic thermodynamic incompatibility between PS and poly(ethylene‐*ran*‐butylene) (EB) blocks (Figure S2). Consequently, a spatially deterministic multiscale composite was successfully fabricated and composed of microscale porous morphology and nanoscale microphase‐separated domains (Figure 2a). The formation of microphase‐separated structures in SEBS is mainly governed by thermodynamic parameters of the system, including the relative volume fraction of each block, total degree of polymerization, and the Flory‐Huggins interaction parameter (*χ*) [37, 38]. By adjusting the block ratio or molecular weight, the morphology and periodicity of the assembled structures and characteristic size of the nanoscale domains can be precisely controlled, which provides a structural basis for tuning the optical and mechanical properties of materials [39, 40]. As shown in Figure 2b, pristine SEBS exhibited characteristic scattering peaks in the small‐angle X‐ray scattering (SAXS) profile, corresponding to the microphase‐separated structure formed by PS domains in EB matrix. The appearance of a shoulder peak at roughly √3*q* and absence of high‐order peaks suggest some level of microphase separation (possibly in spherical or cylindrical morphology) without long‐range order [41]. After adding DOPO, the similar shape of the SAXS profiles corroborated the composite maintained similar microphase‐separated morphologies. Even at relatively high DOPO loading, the characteristic SAXS peak remains clearly visible, indicating that the intrinsic microphase‐separated structure of SEBS was preserved. The primary scattering peak gradually shifted toward lower *q* values with increased DOPO loadings, indicating an increase in domain spacing calculated according to the relationship *d = 2π/q* (Figure S3). The slightly increased domain spacing suggests DOPO molecules may preferentially locate in one phase and induce domain swelling [42]. Meanwhile, the relative intensity of the scattering peak decreased with increasing DOPO content. This behavior may originate from the plasticizing effect of DOPO, which alters the interaction between PS and EB blocks and weakens the microphase separation in the SEBS [43]. As illustrated in the 2D SAXS patterns, all samples displayed uniform isotropic scattering rings, indicating the microphase‐separated domains remained randomly oriented without preferential alignment.

Fourier transform infrared spectroscopy (FTIR) (Figure 2c) confirms the successful incorporation of DOPO into SEBS matrix. Compared to pristine SEBS, the SEDO exhibits several new absorption bands within 750–1250 cm^−1^, which are assigned to the characteristic stretching vibrations of P═O and P─O─C groups in DOPO. The preserve of intrinsic SEBS characteristic peaks indicated that DOPO was physically blended instead of forming chemical bonding. X‐ray diffraction (XRD) results show that crystalline DOPO exhibits multiple sharp diffraction peaks. In contrast, no characteristic diffraction peaks of DOPO are observed in the SEDO, where only a broad peak originating from the amorphous polymer matrix is detected (Figure S4). This result suggests that DOPO does not form independent crystals in the SEBS matrix but was dispersed in the polymer matrix. The dispersion of DOPO in the polymer matrix was further examined by scanning electron microscopy (SEM) combined with energy‐dispersive X‐ray spectroscopy (EDS). As shown in Figure 2d, the SEM image displays a relatively uniform surface morphology without particle aggregation, indicating decent dispersity of DOPO in the SEBS matrix. The corresponding EDS elemental mapping shows a homogeneous distribution of phosphorus (P) throughout the observed region. However, since the microphase‐separated structure of SEBS mainly exists at the nanoscale, SEM cannot directly distinguish whether DOPO is located in the PS domains or EB matrix. To further clarify the distribution of DOPO in SEBS, differential scanning calorimetry (DSC) was employed to investigate the thermal transition behavior of the composites. Microphase‐separated SEBS typically exhibits two distinct glass transition temperatures (*T_g_*), corresponding to the EB and PS, respectively. As shown in Figure 2e,f, the *T_g_* of the pristine EB is about ‐54.4°C, while the *T_g_* of the pristine PS is about 89.0°C. After the introduction of DOPO, the *T_g_* of the EB phase remains nearly unchanged, with all samples maintaining *T_g_* values around ‐55°C, indicating the addition of DOPO didn't change the *T_g_* of EB. In contrast, the *T_g_* of the PS decreases significantly with increasing DOPO content, from 89.0°C of pristine SEBS to 77.5°C and 59.8°C, respectively. The reduction in *T_g_* of the PS suggests that DOPO molecules may preferentially locate in PS domains and produce pronounced plasticization effect, leading to enhanced chain mobility in the PS phase [44]. In addition, the melting transition of crystalline DOPO disappears after incorporation into SEBS, consistent with the XRD results and indicating the absence of a detectable independently crystallized DOPO phase (Figure S5). Together with the accompanying microphase structural evolution revealed by SAXS, these thermal and structural results provide complementary evidence supporting the preferential partitioning of DOPO into the PS domains. This selective distribution is likely due to the higher structural similarity and compatibility between DOPO and the aromatic PS segments.

### 2.2 Molecular Vibrational and Microscopic Spatial Engineering for Enhanced Atmospheric Window Emissivity

**FIGURE 3:**
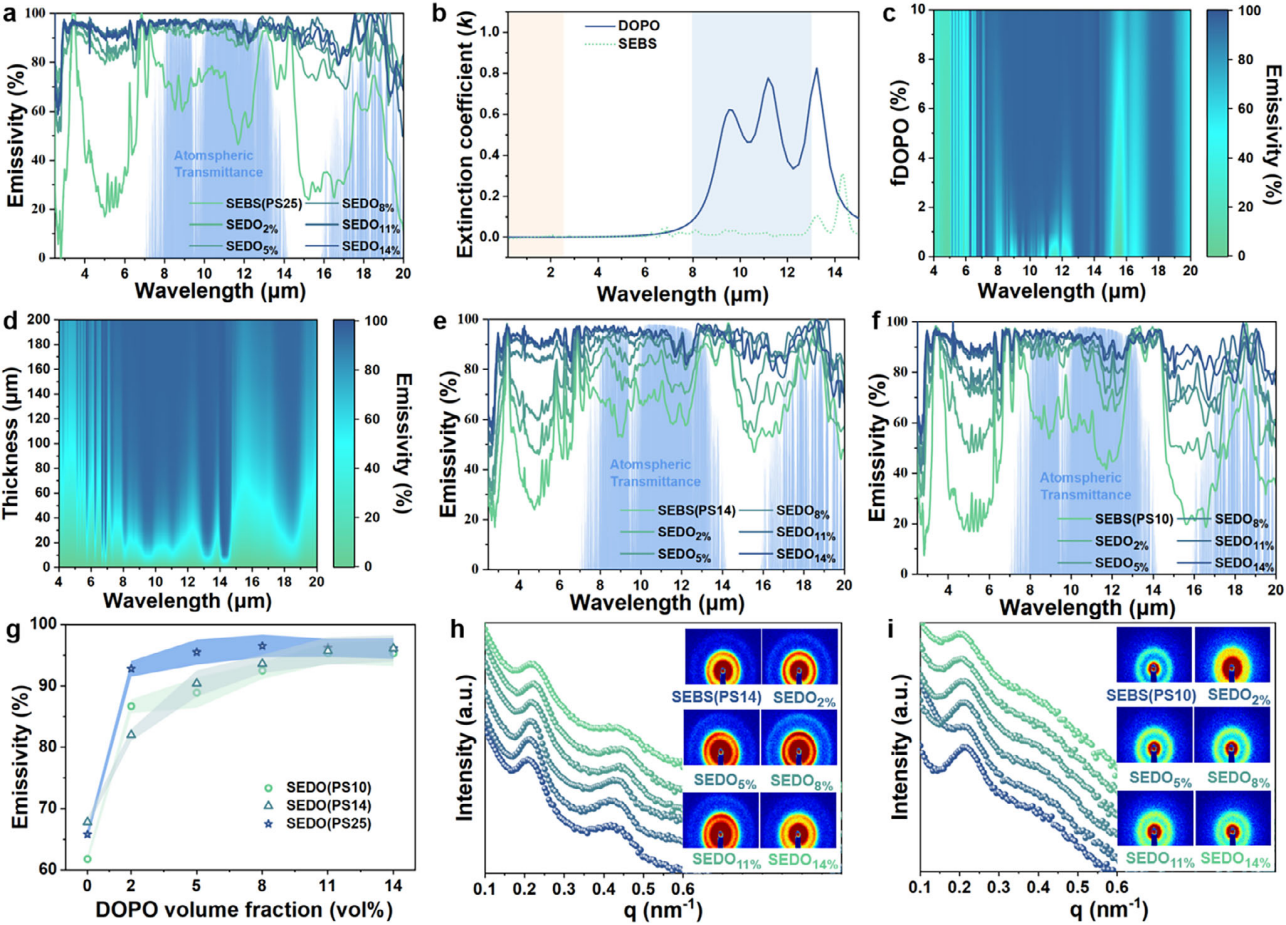
Molecular vibrations and microphase separation engineering for the modulation of atmospheric window emissivity in SEDO. (a) Infrared emissivity spectra of SEDO with different DOPO loadings. (b) Extinction coefficient of DOPO and SEDO. (c) Effect of DOPO loading on the infrared emissivity of SEDO. (d) Effect of film thickness on the infrared emissivity of SEDO. (e) Infrared emissivity spectra of SEDO with different DOPO loadings at a PS content of 14%. (f) Infrared emissivity spectra of SEDO with different DOPO loadings at a PS content of 10%. (g) Effect of PS content on the infrared emissivity of SEDO. (h, i) SAXS patterns of SEDO with different DOPO loadings at PS contents of 14% and 10%, respectively.

To achieve stable radiative cooling performance under practical operating conditions, materials need to endow desired optical properties in both the solar spectrum and the atmospheric window. As shown in Figure 3a and Figure S6, pristine SEBS exhibits an average infrared emissivity of about 65.8% throughout the 8–13 µm atmospheric window. With the introduction of DOPO, the infrared emission is significantly enhanced. The emissivity reaches as high as 96.5% when the DOPO content increases to 8%. The enhancement in infrared emissivity mainly originates from the incorporation of P═O and P─O─C functional groups from DOPO molecules, which possess strong characteristic vibrational absorption in the 8–13 µm range. These intense molecular vibrations generate multiple pronounced extinction peaks within the atmospheric window, thereby increasing the infrared absorption capability of the material in this wavelength range (Figure 3b and Figure S7). According to Kirchhoff's law, the infrared absorptivity equals to the emissivity under thermal equilibrium. Therefore, the enhanced molecular vibrational absorption effectively improves the infrared radiative capability of the composites. To further evaluate the influence of DOPO incorporation on the overall infrared radiative response, the emissivity of the composites was simulated using a Monte Carlo radiative transfer model [45]. In this model, the optical contribution of DOPO was treated using a spatially homogenized approximation. The simulation reproduces the experimentally observed overall increase in infrared emissivity with increasing DOPO loading (Figure 3c and Figure S8), although some discrepancies remain in the detailed spectral shape and relative peak intensities. These discrepancies may arise from the homogenized treatment of DOPO and the simplified representation of the complex porous morphology. In addition, the effect of film thickness on infrared emissivity was simulated at a DOPO loading of 5 vol% (Figure 3d). The emissivity in the 8–13 µm atmospheric window gradually increases with increasing film thickness and eventually reaches a stable value. This behavior can be attributed to the longer effective optical path length for infrared radiation in thicker films, which enhances molecular vibrational absorption and thus improves the overall radiative emission capability, providing an important optical basis for achieving stable and efficient radiative cooling.

However, achieving infrared emissivity higher than 95% at low DOPO loading remains challenging. At low DOPO loading, the contribution of infrared‐active groups in DOPO to thermal radiation is limited by the spatial distribution and local concentration within the SEBS matrix. In addition to increasing the DOPO loading, regulating its microscopic and spatial distribution may provide an effective alternative for improving infrared emission. Since DOPO preferentially distributes in the PS domains of SEBS, the spatial distribution of DOPO in the composite can be optimized by adjusting the microphase‐separated structure of the block copolymer. To verify this effect, the influence of different block ratios, corresponding to PS contents of 10%, 14%, and 25%, on the infrared emission performance of the SEDO was systematically investigated. As shown in Figure 3e,f and Figure S9, with same DOPO loading of 5 vol%, the infrared emissivity in the 8–13 µm atmospheric window proportionally increases with increasing PS content. The emissivities in the atmospheric window for the SEDO(PS10), SEDO(PS14), and SEDO(PS25) are 88.9%, 90.4%, and 95.5%, respectively. Notably, when the PS content reaches 25%, a high emissivity of 95.5% can be achieved with only 5 vol% DOPO (Figure 3g), demonstrating that regulating the block ratio enables excellent infrared radiative performance at relatively low additive loading.

To clarify the influence of microphase‐separated structures on infrared emission performance, the microphase morphology of SEDO(PS10), SEDO(PS14), and SEDO(PS25) with different DOPO loadings was characterized. The PS contents were determined from the integral ratios of the ^1^H NMR spectra (Figure S10). All samples exhibit similar characteristic scattering peaks together with uniform isotropic scattering rings, validating that the typical microphase‐separated structure composed of PS nanodomains in the EB matrix is preserved (Figure 3h,i). After thermal annealing, the second‐ and third‐order scattering peaks (*q*_2_ and *q*_3_) become more clearly resolved. According to the peak positions, the PS10 affords a spherical morphology, while the SEDO(PS14) and SEDO(PS25) show cylindrical microstructures (Figure S11). Notably, the SEDO(PS14) and SEDO(PS25) display significantly higher infrared emissivity than the SEDO(PS10). A similar trend is also observed in the neat SEBS systems, where SEBS(PS14) and SEBS(PS25) with cylindrical morphology exhibit higher infrared emissivity than the spherical SEBS(PS10) (Figure 3g), further supporting the critical role of microphase morphology in determining infrared emission performance. In addition, increasing PS content affects the characteristic domain size. Based on the structural parameters of the cylindrical phase, the SEDO(PS25) possesses larger cylindrical domain dimensions than SEDO(PS14). This enrichment enhances the vibrational absorption of infrared‐active groups such as P═O and P─O─C, thereby improving infrared emission within the atmospheric window. Therefore, by regulating the microphase‐separated structure of the block copolymer composite, the spatial distribution of DOPO and the light‐matter interaction within the composite can be effectively tuned, enabling augmented infrared emissivity in the atmospheric window at relatively low additive loading. It should be noted that further increasing the PS fraction may modify the infrared response but can also drive the SEBS microphase morphology from cylindrical toward lamellar structures, accompanied by pronounced changes in mechanical behavior. Therefore, among the SEBS compositions investigated in this work, SEDO(PS25) was selected for subsequent investigation.

### 2.3 Macroscopic Phase Segregation for Strengthened Solar Reflectivity

**FIGURE 4:**
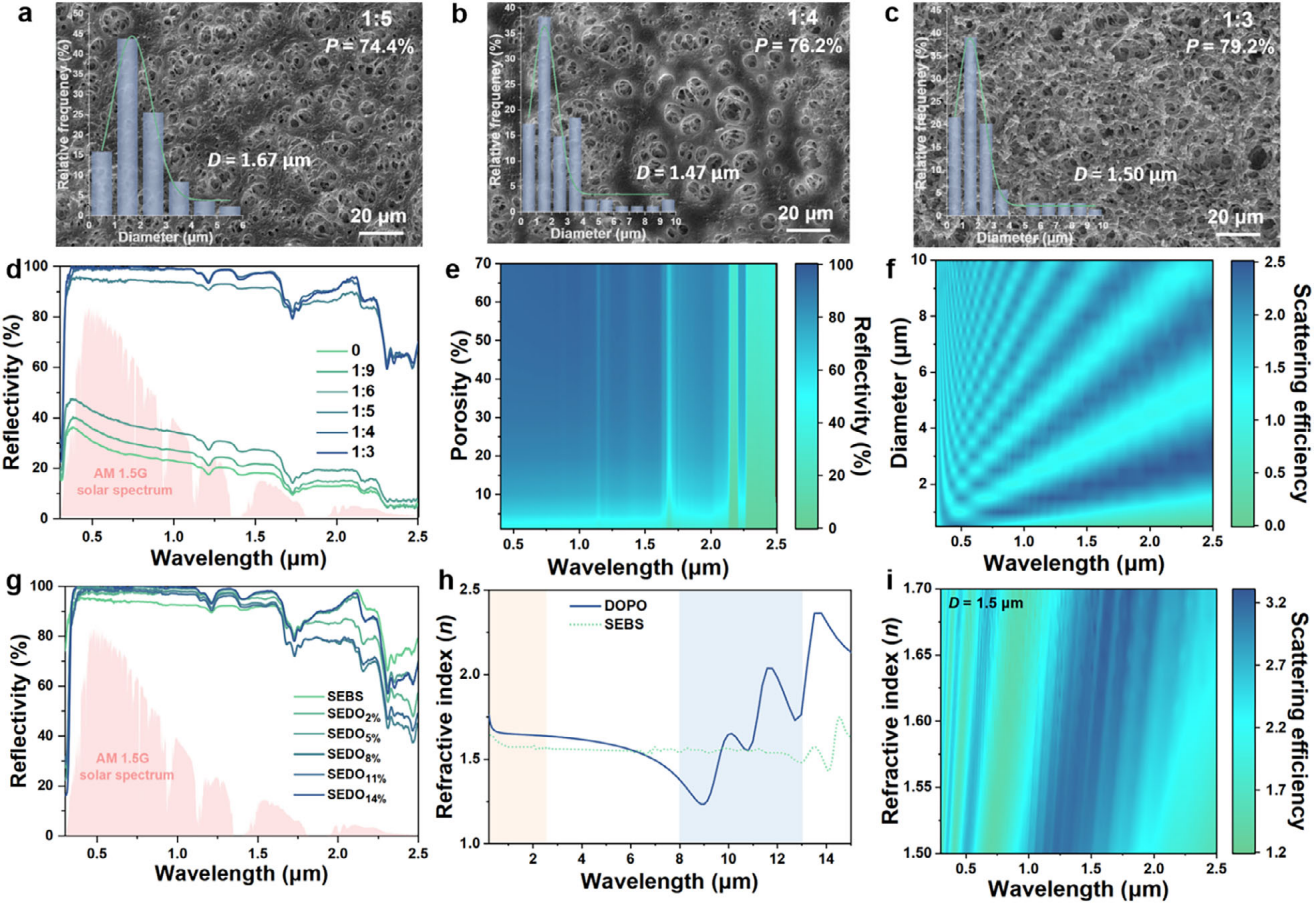
Macroscopic phase segregation for tailoring solar reflectivity in SEDO. (a‐c) SEM images of SEDO prepared with different solvent‐to‐nonsolvent volume ratios; insets show the corresponding pore‐size distribution histograms. (d) Solar reflectivity spectra of SEDO prepared with different solvent‐to‐nonsolvent volume ratios. (e) Effect of porosity on the solar reflectivity. (f) Effect of pore size on the scattering efficiency. (g) Solar reflectivity spectra of SEDO with different DOPO loadings. (h) Refractive index of SEBS and DOPO. (i) Effect of refractive index on the scattering efficiency.

It is important to note that stable radiative cooling performance also requires high reflectivity and scattering in the solar spectrum to effectively suppress heat input from solar irradiation. Therefore, macroscopic phase segregation of SEDO was regulated to construct porous structure with optimized porosity and pore‐size distribution. By adjusting the volume ratio of solvent to nonsolvent, the porosity could be tailored from 0 up to 79.2%. As shown in Figure 4a–c and Figure S12, the porosity gradually increases with increasing nonsolvent content. When the solvent‐to‐nonsolvent volume ratio lies between 1:5 and 1:3, high porosity exceeding 70% were obtained, accompanied by different pore‐size distributions. In particular, a three‐dimensionally interconnected porous structure was achieved at a solvent‐to‐nonsolvent volume ratio of 1:3, which can significantly enhance multiple scattering of light and thereby improving the overall reflectivity. Correspondingly, when the solvent‐to‐nonsolvent volume ratios are 1:5, 1:4, and 1:3, the average solar reflectivity of as‐prepared SEDO reaches 92.4%, 96.9%, and 97.2%, respectively (Figure 4d). This trend is further supported by the simulation calculations, which show a progressive enhancement in solar reflectivity with increasing porosity (Figure 4e; Figure S13). In addition, the solar reflectivity gradually increases with increasing film thickness and eventually reaches to a plateau (Figure S14). Longer optical propagation path in thickened porous structures enhances multiple scattering and improves the overall reflectivity. Finite‐difference time‐domain (FDTD) simulations were performed to evaluate the scattering efficiency associated with different pore sizes. As shown in Figure 4f, pores with characteristic sizes of 0.5–2 µm exhibit strong scattering in the visible region, whereas larger pores in the range of 2–10 µm contribute more effectively to scattering in the near‐infrared region. Therefore, the broad pore‐size distribution provides scattering centers over different wavelength ranges, enabling broadband scattering across the solar spectrum. Meanwhile, the high porosity and three‐dimensionally interconnected porous network introduce abundant polymer/air interfaces and extend the effective optical propagation path through repeated scattering events, thereby further enhancing the overall scattering intensity. Consequently, the broad pore‐size distribution and three‐dimensionally interconnected porous structure of SEDO synergistically enable efficient broadband solar scattering, thereby significantly enhancing the overall solar reflectivity.

While the porous architecture provides the primary structural basis for broadband solar scattering, the scattering strength at the solid/air interfaces can be further regulated by their refractive‐index contrast. As shown in Figure 4g, the solar reflectivity of SEDO gradually increases with increasing DOPO loading, rising from 92.4% for pristine porous SEBS to 97.7%. This additional enhancement is attributed to the relatively high refractive index of DOPO (n ≈ 1.645) compared with that of the SEBS matrix (n ≈ 1.50) (Figure 4h). Incorporation of DOPO therefore increases the optical contrast between the solid framework and the air voids (n ≈ 1), strengthening light scattering at the abundant interfaces generated by the porous architecture. In contrast, solely tuning the porous structure of pristine SEBS through macroscopic phase segregation is insufficient to achieve solar reflectivity exceeding 95% (Figure S15). Simulation results also reveal that an increased refractive index mismatch significantly enhances the scattering efficiency, thereby improving the solar reflection performance (Figure 4i). Considering that 5 vol% DOPO already enables simultaneously high solar reflectivity and atmospheric window emissivity, while further increasing the DOPO loading provides only limited additional improvement in the overall optical performance, SEDO(PS25) with a solvent‐to‐nonsolvent volume ratio of 1:3 and a DOPO loading of 5 vol% was selected for subsequent investigations. This composition exhibits a solar reflectivity of 97.2% and an infrared emissivity of 95.5%, representing a favorable balance between high radiative cooling performance and moderate DOPO loading.

To evaluate the radiative cooling capability of the SEDO, the net cooling power was calculated under different non‐radiative heat transfer coefficients (*h*_c_). The solar irradiance was set to 1000 W m^−2^, while the ambient temperature was fixed at 25°C. The results indicate the SEDO can achieve net cooling powers of 104.3 W m^−2^ during daytime and 132.3 W m^−2^ at nighttime (Figure S16). Under daytime conditions, when *h*_c_ = 6 W m^−2^ K^−1^, a cooling temperature of ∼13°C can be achieved. Even under relatively strong conductive and convective conditions (i.e., *h*_c_ = 12 W m^−2^ K^−1^), the SEDO still maintains a sub‐ambient cooling of ∼7°C, demonstrating its excellent capability to deliver stable radiative cooling performance under complex outdoor environments.

### 2.4 Stable Outdoor Passive Radiative Cooling Performance

**FIGURE 5:**
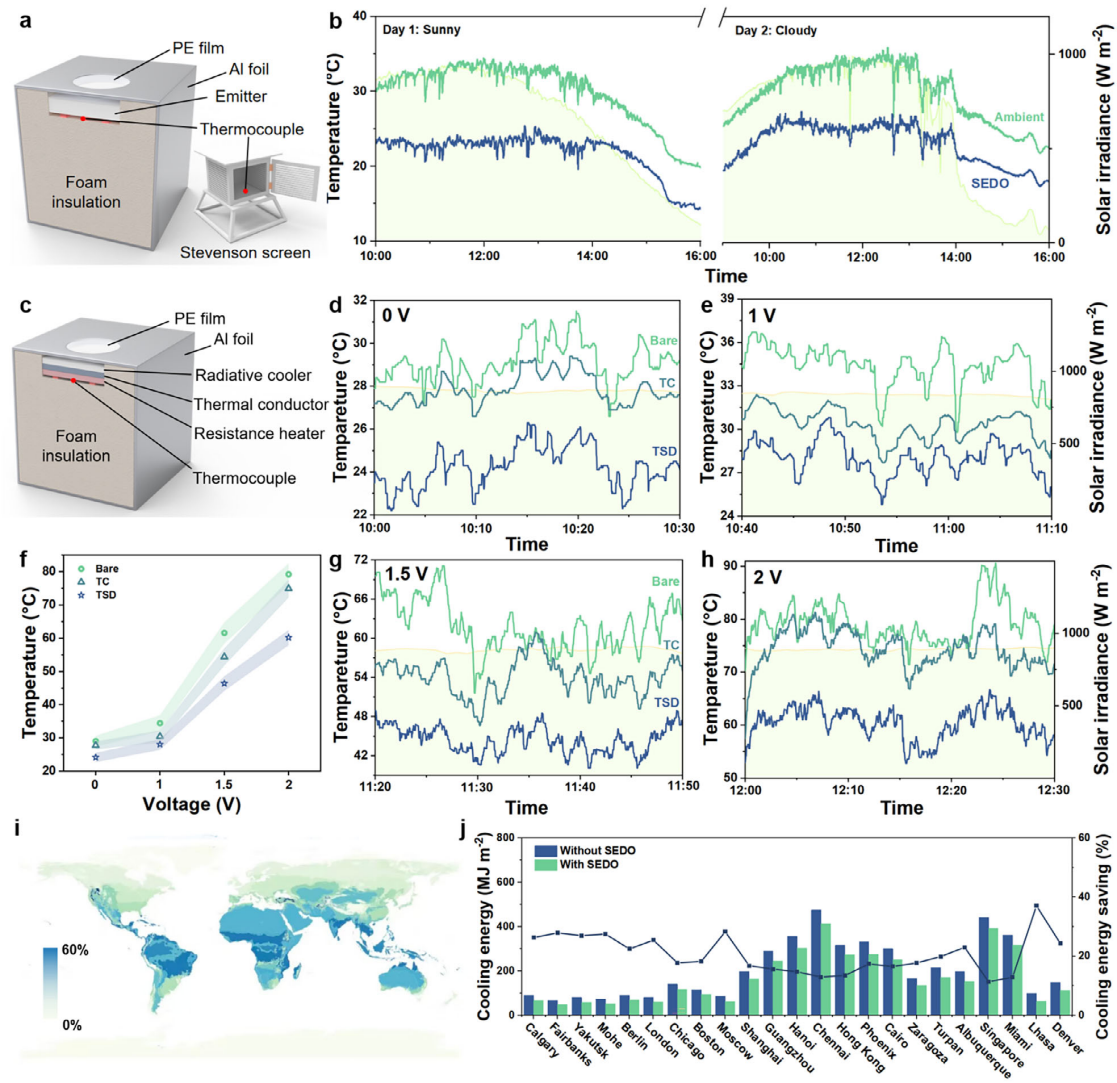
Outdoor cooling performance and thermal load adaptability of SEDO. (a) Schematic illustration of the outdoor thermal performance test setup. (b) Outdoor cooling performance of SEDO on a sunny day (October 28, 2025) and a cloudy day (October 29, 2025). (c) Schematic illustration of the outdoor thermal performance test with a tunable external heat source. (d‐h) Outdoor cooling performance of TSD and TC under different applied voltages. (i, j) Comparison of cooling energy consumption and cooling energy saving enabled by SEDO across major cities worldwide.

To evaluate the practical radiative cooling performance of the SEDO, an outdoor thermal performance testing platform was constructed. The measurements were conducted on a rooftop in Nanjing, China (32°53′ N, 118°48′ E) in October. The testing device was placed on a tiled supporting platform to minimize heat conduction from the surrounding environment. Meanwhile, the setup was sealed with a low‐density polyethylene film to suppress air convection interference. The ambient temperature was continuously monitored using a thermocouple housed in a louvered radiation shield (Figure 5a). Under clear‐sky conditions, the average solar irradiance was measured to be 666.8 W m^−2^ during the test period, with a relative humidity of 44.3% and a wind speed of 0.64 m s^−1^. The SEDO exhibited a temperature reduction of ∼8.1°C below the ambient temperature. Under partly cloudy conditions, with an average solar irradiance of 658.7 W m^−2^, relative humidity of 55.9%, and wind speed of 1.09 m s^−1^, the SEDO achieved a sub‐ambient cooling of ∼6.3°C (Figure 5b and Figure S17). The excellent cooling performance is attributed to the decoupled spectral response enabled by the multiscale structural design of the SEDO, allowing the material to simultaneously achieve a high average solar reflectivity of 97.2% and an infrared emissivity of 95.5%, even at relatively low DOPO loading. The experimentally measured outdoor cooling results show good agreement with the theoretically predicted cooling performance, corroborating the high‐efficiency radiative cooling capability of the SEDO under practical environmental conditions.

**FIGURE 6:**
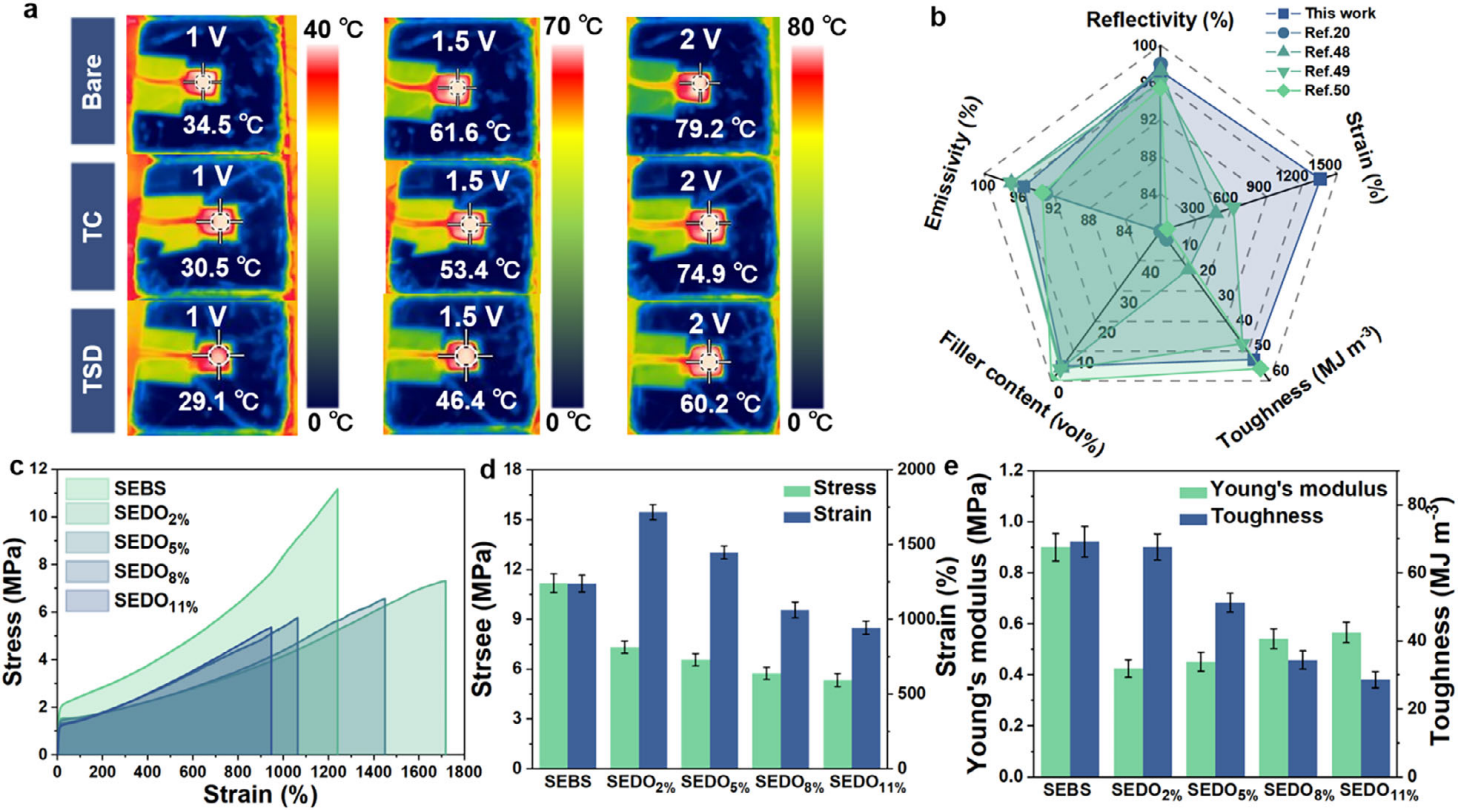
Thermal dissipation and mechanical properties of SEDO. (a) Infrared thermal images of the TSD, TC, and bare heater surfaces under different input voltage conditions. (b) Radar plot comparison of the SEDO with representative advanced radiative cooling materials. (c) Stress‐strain curves of SEDO with different DOPO loadings. (d) Comparison of stress and strain of SEDO at different DOPO loadings. (e) Young's modulus and toughness of SEDO at different DOPO loadings.

In practical radiative cooling applications, materials may be subjected to additional thermal loads from heat‐generating objects, such as vehicles, the human body, or electronic devices, which places additional demands on their heat‐dissipation capability. To evaluate whether the experimentally optimized SEDO could maintain effective thermal management under varying external heat inputs, an outdoor thermal performance testing platform with an adjustable external heat source was constructed and tested under direct solar irradiation (Figure 5c). The testing platform employed a metallic resistive heating unit as the external heat source, where the input power was regulated by adjusting the applied voltage using a direct current power supply. The heat source was coupled to the SEDO through a thermally conductive tape (TC), thereby forming a conduction‐radiation cooler (TSD). In such configuration, the heat generated by the source was first transferred to the surface of SEDO through the conductive layer and subsequently dissipated to the surrounding environment via infrared radiation. As shown in Figure 5d–h, and Figure 6a, the TSD consistently exhibits lower temperatures than TC alone under the investigated input voltage conditions. When the applied voltages were 0, 1, 1.5, and 2 V, the TSD achieved temperature reductions of ∼3.6, ∼2.4, ∼8, and ∼14.7°C, respectively, relative to TC. Although the temperature difference varies with the applied heat input, the optimized SEDO consistently provides an additional cooling effect over the investigated range, demonstrating its capability to dissipate externally introduced heat through thermal radiation. At low input power, the temperature reduction is mainly attributed to the high solar reflectivity of TSD, which effectively suppresses solar heat gain. As the input power increases and the surface temperature rises, the radiative heat flux increases according to the Stefan–Boltzmann law, thereby enhancing the contribution of radiative heat dissipation. Benefiting from the synergistic effects of high solar reflectivity and high infrared emissivity, the TSD simultaneously suppresses solar heat absorption and enhances infrared radiative heat dissipation, thereby exhibiting more pronounced cooling performance under high input power conditions (Figure S18).

Additional thermal‐load measurements were further performed to examine the effects of PS content and DOPO loading under identical external heat inputs (Figure S19). For SEDO containing 5 vol% DOPO, the temperature differences among SEDO(PS10), SEDO(PS14), and SEDO(PS25) became more pronounced with increasing heat input. At 2 V, their equilibrium temperatures were 66.2, 64.8, and 60.2°C, respectively, consistent with the enhanced infrared radiative capability of SEDO(PS25) at elevated temperatures. The influence of DOPO loading was further evaluated for SEDO(PS25). Increasing the DOPO loading from 2 to 5 vol% reduced the equilibrium temperature from 63.7 to 60.2°C at 2 V, whereas a further increase to 8 vol% resulted in only a marginal decrease to 59.6°C. Interestingly, SEDO with 5 vol% DOPO exhibited slightly lower temperatures at low heat inputs, whereas the 8 vol% sample showed a marginal thermal advantage as the heat input increased. This behavior can be attributed to the competing effects of solar reflection and infrared emission: further DOPO incorporation enhances infrared emissivity but is accompanied by a decrease in solar reflectivity.

To evaluate the building energy‐saving potential of the experimentally optimized SEDO under different climatic conditions, annual cooling energy consumption was simulated using a standardized building model in EnergyPlus (Figure S20). Across 23 representative cities spanning diverse climate zones, replacing conventional envelope materials with the SEDO composite results in a substantial reduction in cooling energy demand (Figure 5i). On average, a 20.6% reduction is achieved globally (Figure 5j), with more pronounced savings observed in tropical and subtropical regions where cooling loads dominate. In hot‐arid city such as Lhasa, the reduction exceeds 35%. In temperate climates such as Berlin and London, savings remain above 20%. Additional thickness‐dependent simulations further showed that increasing the SEDO thickness leads to only a modest improvement in building cooling‐energy savings, indicating that once sufficient thickness is reached to achieve the optimized optical performance, further increasing the film thickness provides only limited additional benefits for building energy conservation (Figure S21). These results demonstrate the strong potential of the SEDO coating for large‐scale building energy savings, particularly in cooling‐dominated climates.

Notably, SEDO achieves an excellent combination of optical performance and flexibility at a relatively low filler loading, effectively overcoming the typical trade‐off between heat dissipation and mechanical integrity. As shown in Figure 6c, pristine SEBS exhibits typical elastomeric behavior with high elongation at break and decent tensile strength of 11.2 ± 0.6 MPa and 1239.6 ± 55.5%, which originates from the microphase‐separated structure where PS nanodomains act as physical cross‐linking points within the EB matrix. The mechanical properties of the composites change with the introduction of DOPO. Overall, the fracture stress gradually decreases with increasing DOPO content, while the elongation at break first increases at low loading and then decreases at higher loading levels (Figure 6d). Specifically, the composite exhibits the highest elongation at break of 1717.1 ± 50.7% with at a DOPO content of 2 vol%, whereas further increasing the DOPO loading leads to a gradual reduction in elongation at break. Meanwhile, the toughness of the material also decreases with increasing DOPO content (Figure 6e). Nevertheless, at a DOPO content of 5 vol%, the composite still maintains a toughness of ∼51.2 MJ m^−3^ together with high stretchability, indicating that SEDO retains substantial deformation capability after DOPO incorporation. The variation in mechanical behavior is closely related to the interaction of DOPO with the SEBS matrix. The plasticization effect of DOPO reduces the constraint of the PS domains, making structural rearrangement easier during stretching and thereby leading to a gradual decrease in fracture strength [46, 47]. On the other hand, at low DOPO loading, moderate plasticization increases chain mobility, allowing the material to sustain larger deformation, which results in higher elongation at break at 2 vol% and 5 vol% loading. However, excessive DOPO may act as a stress concentrator, resulting in a reduction in elongation at break. Therefore, the mechanical advantage of SEDO lies primarily in its excellent flexibility and large deformation capability rather than exceptionally high tensile strength or toughness. More importantly, SEDO simultaneously achieves high solar reflectivity and infrared emissivity while retaining the flexibility of the SEBS‐based system, demonstrating a favorable combination of radiative cooling performance and mechanical flexibility (Figure 6b and Table S1) [20, 48, 49, 50, 51, 52, 53, 54, 55]. This comparison highlights the favorable integration of high radiative cooling performance, low additive loading, and mechanical flexibility enabled by the multiscale phase‐segregation strategy.

The excellent stretchability of SEDO provides additional advantages for flexible and deformable radiative cooling applications, such as integration onto curved substrates, protective films, and deployable outdoor thermal‐management structures. In these scenarios, the elastomeric nature of SEBS enables the cooling layer to better tolerate mechanical deformation while maintaining structural integrity. To further evaluate the mechanical response of SEDO under repeated deformation, cyclic tensile tests were performed at 100% strain. As shown in Figure S22a,b, the SEDO composite withstood 50 consecutive loading‐unloading cycles at 100% strain without mechanical failure, demonstrating its ability to tolerate repeated large deformation. More importantly, the solar reflectivity and atmospheric window emissivity were well preserved after cyclic stretching, with values of 96.9% and 95.4%, respectively, compared with 97.2% and 95.5% for the pristine sample (Figure S22c,d). These results demonstrate that SEDO retains its radiative cooling‐related optical properties under repeated mechanical deformation. These results further support the selection of 5 vol% DOPO as the optimized composition. Although increasing the DOPO loading to 8 vol% provides only a marginal enhancement in infrared emissivity, it is accompanied by reductions in elongation at break and toughness. Therefore, 5 vol% DOPO provides a favorable balance between radiative cooling performance and mechanical flexibility.

The SEDO also processes excellent thermal stability and long‐term durability. Thermogravimetric analysis (TGA) and its derivative thermogravimetric (DTG) curves reveal that no significant decomposition occurs below ∼200°C (Figure S23). The actual DOPO loading, calculated from the TGA mass loss, shows good agreement with the theoretical feeding amount, suggesting that DOPO is effectively embedded within the SEBS matrix during fabrication without noticeable precipitation or solvent‐induced loss. At a DOPO loading of 5 vol%, the SEDO demonstrates significantly enhanced flame‐retardant performance compared to pristine SEBS (Figure S24). Cone calorimetry results show that the peak heat release rate (PHRR) and total heat release (THR) decrease by 33.2% and 31.8%, respectively, while the CO_2_ release is reduced by 35.1%. This performance improvement is primarily attributed to the phosphorus‐based flame‐retardant mechanism of DOPO. During thermal decomposition, DOPO generates phosphorus‐containing radicals (e.g., PO· and PO_2_·), which effectively capture reactive radicals such as H· and OH· in the combustion process, thereby suppressing gas‐phase chain reactions [56, 57, 58]. Meanwhile, phosphorus‐containing intermediate species promote the formation of a stable char layer in the condensed phase, which reduces heat release and inhibits the diffusion of combustible gases and oxygen. In addition, the SEDO exhibits good environmental stability. After continuous outdoor exposure for 720 h, no noticeable decrease is observed in either the average solar reflectivity or infrared emissivity, demonstrating the stable optical performance of SEDO under prolonged outdoor conditions (Figure S25). Considering that long‐term ultraviolet (UV) irradiation may induce photoaging of polymeric materials and consequently affect their optical properties, the UV stability of SEDO was further evaluated. After 168 h of UV irradiation, the solar reflectivity and infrared emissivity remain nearly unchanged compared with their initial values (Figure S26), demonstrating the good resistance of SEDO to UV‐induced optical degradation. These results indicate that SEDO can maintain stable optical properties under both prolonged outdoor exposure and UV irradiation, supporting its potential for long‐term outdoor radiative cooling applications. SAXS at elevated temperature confirms that the nanoscale microphase structure remains stable without obvious structural transition until 120°C during heating (Figure S27). Meanwhile, no characteristic diffraction peaks associated with DOPO appear in the XRD patterns after heating at elevated temperature, suggesting DOPO maintains good dispersion within the SEBS matrix without crystallization or phase separation (Figure S28). The combination of excellent thermal stability, flame‐retardant capability, and environmental durability enables the SEDO to maintain long‐term stable thermal management performance in practical radiative cooling applications. From a manufacturing perspective, the solution‐based phase‐segregation strategy may offer potential compatibility with large‐area coating processes, although its applicability to continuous manufacturing remains to be experimentally validated. Further optimization of processing parameters and the solvent system will be required for future scale‐up.

## 3 Conclusion

In summary, a SEDO block copolymer composite was judiciously designed and crafted by systematic tailoring the macroscopic and microphase segregation, enabling decoupled spectral optimization for passive radiative cooling. Macroscopic phase segregation endowed three‐dimensionally interconnected porous framework that promotes broadband solar scattering, while microphase separation determined the spatial distribution of infrared‐active molecules and rendered efficient thermal emission within the atmospheric window. Benefiting from this multiscale phase segregation structure, the composite achieved high solar reflectivity of 97.2% and high infrared emissivity of 95.5% with only a 5 vol% DOPO additive loading, enabling stable radiative cooling under varying external heat loads. Outdoor tests demonstrated that SEDO achieved a cooling effect of ∼8.1°C below ambient temperature on sunny days and ∼6.3°C on cloudy days. Additionally, the SEDO exhibited excellent mechanical flexibility and decent toughness of ∼51.2 MJ m^−3^, overcoming the typical trade‐off between heat dissipation and mechanical integrity in composite structure. This multiscale phase segregation strategy provides an effective pathway for correlating optical properties with structures across distinct length scales and offers a promising approach for stable radiative cooling in complex thermal‐management environments.

## 4 Experimental Section

### 4.1 Materials

Polystyrene‐block‐poly(ethylene‐ran‐butylene)‐block‐polystyrene (SEBS) with different polystyrene (PS) contents were supplied by Asahi Kasei Corporation (Tokyo, Japan). 9,10‐Dihydro‐9‐oxa‐10‐phosphaphenanthrene‐10‐oxide (DOPO, ≥99.8%) was purchased from Sanko Co., Ltd. (Kurume, Japan). Polystyrene (PS, M_w_ ≈ 192,000) was obtained from Sigma‐Aldrich (St. Louis, MO, USA). Chloroform (≥99%) and isopropanol (IPA, ≥99.7%) were purchased from Sinopharm Chemical Reagent Co., Ltd. (Shanghai, China).

### 4.2 Preparation of Block Copolymer Composite (SEDO)

SEBS and DOPO were dissolved in 20 mL of chloroform and magnetically stirred at room temperature for 6 h until complete dissolution was achieved. Subsequently, a predetermined amount of isopropanol was added, followed by ultrasonication for 5 min to ensure homogeneous mixing. The resulting solution was then cast into a customized polytetrafluoroethylene mold and placed in a fume hood, where macrophase separation and block copolymer self‐assembly occurred simultaneously, leading to the formation of block copolymer composite (SEDO). SEDO with different DOPO loadings (2, 5, 8, 11, and 14 vol%) were prepared for comparison and denoted as SEDO_2%_, SEDO_5%_, SEDO_8%_, SEDO_11%_ and SEDO_14%_, respectively.

### 4.3 Characterization

The microstructure and elemental distribution of the polymers were examined using field‐emission scanning electron microscopy (SEM, FEI Nova NanoSEM 450, USA). The pore size distribution was statistically analyzed using Image J software (Image J). Fourier transform infrared spectra were recorded in attenuated total reflectance mode (ATR) using an FTIR spectrometer (FTIR, Thermo Scientific Nicolet iS50, USA). The crystalline structures of the samples at room and elevated temperatures were characterized by in situ X‐ray diffraction (XRD, Rigaku SmartLab, Japan) with an operating voltage of 40 kV and current of 40 mA. The small angle scattering peaks and 2D patterns were obtained using small‐angle X‐ray scattering (SAXS, Anton Paar Gmbh point 2.0, Austria). The mechanical properties were measured using a universal testing machine (UTM, MTS HFT‐150D, USA) at a tensile rate of 10 mm/min. The number‐average molecular weight (*M*_n_), weight‐average molecular weight (*M*_w_), and dispersity (Đ) were measured by gel permeation chromatography (GPC, Shimadzu Nexera, Japan) equipped with a refractive index detector. Tetrahydrofuran (THF) was employed as the mobile phase at a flow rate of 1.0 mL/min at 40°C. The molecular weights were calibrated against polystyrene standards. Proton nuclear magnetic resonance (^1^H NMR) spectra were recorded on an NMR spectrometer (NMR, Bruker Avance III 600 MHz, Germany), and the block composition was determined by peak integration. The glass transition temperature (*T_g_*) was measured using differential scanning calorimetry (DSC, Netzsch DSC 214 Polyma, Germany), after eliminating the thermal history of the samples. Thermogravimetric analysis was conducted under a nitrogen atmosphere using a thermogravimetric analyzer (TGA, Netzsch TG 209 F3, Germany) at a heating rate of 10°C/min up to 800°C to evaluate thermal stability. The flame‐retardant performance was assessed using a cone calorimeter (VOUCH 6810 Cone Calorimeter, China), with sample dimensions of 10 × 10 cm. The UV aging test was conducted by exposing the sample to UV lamp radiation (50 W m^−2^) at a distance of 10 cm. After 168 h of UV irradiation, spectral characterization was performed.

### 4.4 Optical Properties

The refractive index (*n*) and extinction coefficient (*k*) of the materials were determined using a spectroscopic ellipsometer (J.A. Woollam RC2, USA). The solar reflectivity spectra were measured using an UV‐visible‐near‐infrared spectrophotometer equipped with an integrating sphere (UV–Vis‐NIR, Shimadzu UV3600, Japan). The infrared reflectivity (*R*) and transmittance (*T*) in the wavelength range of 2.5‐20 µm were characterized using a Fourier transform infrared spectrometer equipped with a gold integrating sphere (FTIR, Thermo Scientific Nicolet iS50, USA). The emissivity is calculated according to the Kirchhoff's law.

### 4.5 Outdoor Thermal Measurements

Outdoor passive cooling tests were conducted on the rooftop of a teaching building at Jiulonghu Campus, Southeast University (32°53′ N, 118°48′ E). The experimental setup consisted of a polystyrene foam box (30 × 30 × 30 cm) with a central cavity (10 × 10 × 1 cm) on the top surface for placing the samples. To minimize direct solar irradiation, the exterior of the setup was wrapped with aluminum foil. The top was sealed with a polyethylene film to effectively suppress undesired heat conduction and convection. A K‐type thermocouple was attached to the backside of each sample to monitor the temperature in real time, while the ambient temperature was recorded by a shaded thermocouple placed inside a louvered radiation shield. Thermal measurements under different external heat input powers were carried out using a similar setup. A metallic resistive heating unit was positioned beneath the sample as an external heat source, and the input power was regulated by adjusting the voltage of a DC power supply. The sample was thermally coupled to the heating element using thermally conductive tape, and temperature measurements were performed under direct solar irradiation. The solar irradiance was recorded in real time using a solar power meter (TES 1333R). The relative humidity and wind speed were monitored using a multifunctional weather station equipped with a louvered radiation shield and an anemometer. The surface temperature characteristics of the samples were recorded using an infrared thermal imaging camera (FLUKE E6, USA).

## Author Contributions

**Mingxin Feng**: conceptualization, methodology, writing – original draft, validation, investigation. **Feiyang Zhou**: conceptualization, methodology, software, data curation, validation. **Haoran Cai**: conceptualization, methodology, validation, data curation. **Wenchao Zhai**: data curation, methodology, validation, formal analysis. **Shuangjiang Feng**: methodology, visualization, software, data curation. **Yanmei Liu**: funding acquisition, supervision, formal analysis, project administration. **Xiaohai Bu**: formal analysis, methodology, visualization, investigation. **Man He**: validation, project administration, supervision. **Dudong Feng**: software, supervision, validation, visualization, resources, writing – review and editing, conceptualization. **Shuang Liang**: writing – review and editing, conceptualization, funding acquisition, writing – original draft, methodology, formal analysis, supervision, data curation. **Yuming Zhou**: writing – review and editing, funding acquisition, conceptualization, project administration, resources, supervision.

## Conflicts of Interest

The authors declare no conflicts of interest.

## Supporting information




**Supporting File**: advs77913‐sup‐0001‐SuppMat.docx.

## Data Availability

The data that support the findings of this study are available from the corresponding author upon reasonable request.
