# Supplementary material for: Architected Spatially Deterministic Multiscale Phase Segregation in Block Copolymer Composites for Augmented Radiative Cooling
Source: Adv Sci (Weinh). 2026 Sep 27:e77913. Online ahead of print. doi: 10.1002/advs.77913 (PMC13616360; doi:10.1002/advs.77913)
Supplement: Supplementary file 1 [file ADVS-9999-e77913-s001.docx]

Supporting Information

**Architected Spatially Deterministic Multiscale Phase Segregation in Block Copolymer Composites for Augmented Radiative Cooling**

Mingxin Feng, Feiyang Zhou, Haoran Cai, Wenchao Zhai, Shuangjiang Feng, Yanmei Liu, Xiaohai Bu, Man He, Dudong Feng*, Shuang Liang*, Yuming Zhou*

**Supplementary Information contains:**

Supplementary Note S1-S3

Supplementary Figures S1-S28

**Note S1: Finite-Difference Time-Domain Simulation**

To investigate the influence of pore structure, pore size, and distribution on the optical properties, finite-difference time-domain (FDTD) simulations were performed using ANSYS FDTD Solutions. The Lorenz-Mie theory, which provides an exact solution to Maxwell’s equations for spherical particles, was employed to accurately describe the interaction between particles and incident light with comparable wavelengths. The refractive index (*n*) and extinction coefficient (*k*) of SEBS and DOPO were obtained from spectroscopic ellipsometry measurements. Perfectly matched layer (PML) boundary conditions were applied to minimize artificial reflections, and open boundary conditions were used to ensure efficient wave absorption. A total-field scattered-field (TFSF) source was adopted as the Mie excitation source, with the wavelength range set from 0.3 μm to 2.5 μm. Cavities were constructed inside a rectangular matrix to model the porous structure, and the relationship between pore diameter and scattering efficiency was calculated. The FDTD simulation was carried out using discrete time steps, where the electromagnetic fields were updated at each step according to Maxwell’s equations. An FDTD cross-section monitor was used to record the scattering efficiency. The scattering efficiency (*Q_sca_*) was obtained by normalizing the scattering cross-section (*C_sca_*), which is expressed as follows:

$Q_{sca}=\frac{C_{sca}}{\pi r^{2}}$	(1)

Where *πr^2^* is the geometrical cross-sectional area of the sphere. The *C_sca_* was calculated by:

$C_{sca}=\frac{2\pi}{k}\sum_{n=1}^{\infty} (2n+1)(\left| a_{n} \right|^{2}+\left| b_{n} \right|^{2})$	(2)

where *k* is the wavevector, and *a_n_* and *b_n_* are the *n^th^* electric mode coefficient and *n^th^* magnetic mode coefficient, respectively. *a_n_* and *b_n_* are evaluated by the Riccati-Bessel functions, *S_n_* and *C_n_*, as follows:

$a_{n}=\frac{mS_{n}(mx){S'}_{n}-S_{n}(x){S'}_{n}(mx)}{mS_{n}(mx){C'}_{n}-S_{n}(x){C'}_{n}(mx)}$	(3)

$b_{n}=\frac{S_{n}(mx){S'}_{n}(x)-mS_{n}(x){S'}_{n}(mx)}{S_{n}(mx){C'}_{n}(x)-mC_{n}(x){S'}_{n}(mx)}$	(4)

where *m* is the relative refractive index.

**Note S2: Simulation of reflectance, transmittance, absorbance and emissivity**

The spectral reflectance, transmittance, absorbance, and emissivity of the porous composite films were simulated using a radiative transfer model for heterogeneous media. The composite film was treated as a multiphase system consisting of SEBS matrix, DOPO fillers, and air pores. The optical response of the system was calculated by combining wavelength-dependent complex refractive indices with photon transport simulation to describe multiple scattering and absorption inside the porous structure.

**S2.1 Effective optical coefficients of the heterogeneous medium**

The composite film was considered as an effective medium composed of polymer matrix, functional fillers, and pores with different volume fractions. The total scattering and absorption coefficients of the medium are expressed as:

$\mu_{s}=\sum_{i} \mu_{s, i}$	(5)

$\mu_{a}=\sum_{i} \mu_{a, i}$	(6)

where *i* represents each component, including SEBS, DOPO, and pores.

The scattering coefficient of each component is written as:

$\mu_{s, i}=\frac{3f_{i}}{2r_{i}}q_{sca, i}$	(7)

and the absorption coefficient is:

$\mu_{a, i}=\frac{3f_{i}}{2r_{i}}(q_{ext, i}-q_{sca, i})$	(8)

where *f_i_* is the volume fraction, *r_i_* is the characteristic size of the scattering unit, q_sca_ and *q_ext_* are scattering and extinction efficiencies, the optical constants *n* and *k* of each component are wavelength dependent.

To account for multiple scattering in high-volume-fraction porous media, a dependent scattering correction factor is introduced:

$C=1+1.5f-0.75f^{2}$	(9)

${\mu'}_{s}=C\mu_{s}$	(10)

${\mu'}_{a}=C\mu_{a}$	(11)

The absorption of the matrix phase is included as:

$\mu_{a, t}={\mu'}_{a}+\frac{4\pi k}{\lambda}(1-f)$	(12)

where *k* is the extinction coefficient of the matrix.

**S2.2 Photon transport simulation**

The propagation of photons inside the composite film was simulated using a Monte Carlo radiative transfer method to obtain spectral reflectance, transmittance, and absorbance.

The photon step length is:

$s=-\frac{\ln\xi}{\mu_{s}+\mu_{a}}$	(13)

where *ξ* is a random number between 0 and 1.

The probability of scattering is:

$P_{s}=\frac{\mu_{s}}{\mu_{s}+\mu_{a}}$	(14)

and the probability of absorption is

$P_{a}=\frac{\mu_{a}}{\mu_{s}+\mu_{a}}$	(15)

By tracing a large number of photons, the spectral reflectance, transmittance, and absorbance are obtained as:

$R_{\lambda}=\frac{N_{ref}}{N_{total}}$	(16)

$T_{\lambda}=\frac{N_{trans}}{N_{total}}$	(17)

$A_{\lambda}=1-R_{\lambda}-T_{\lambda}$	(18)

**S2.3 Solar reflectance, transmittance and absorbance**

The solar-spectrum-averaged optical properties were calculated over the AM1.5 solar spectrum:

$R_{solar}=\frac{\int_{\lambda_{1}}^{\lambda_{2}} R_{\lambda}G_{\lambda}d\lambda}{\int_{\lambda_{1}}^{\lambda_{2}} G_{\lambda}d\lambda}$	(19)

$T_{solar}=\frac{\int_{\lambda_{1}}^{\lambda_{2}} T_{\lambda}G_{\lambda}d\lambda}{\int_{\lambda_{1}}^{\lambda_{2}} G_{\lambda}d\lambda}$	(20)

$A_{solar}=\frac{\int_{\lambda_{1}}^{\lambda_{2}} A_{\lambda}G_{\lambda}d\lambda}{\int_{\lambda_{1}}^{\lambda_{2}} G_{\lambda}d\lambda}$	(21)

where *G_λ_* is the spectral solar irradiance.

**S2.4 Infrared reflectance, transmittance and emissivity**

The infrared optical properties were calculated in the wavelength range of 2.5-25 μm.

According to Kirchhoff’s law of thermal radiation, the spectral emissivity equals absorptivity:

$\varepsilon_{\lambda}=A_{\lambda}=1-R_{\lambda}-T_{\lambda}$	(22)

The emissivity in the atmospheric window (8–13 μm) is

$\varepsilon_{8-13}=\frac{\int_{8}^{13} \varepsilon_{\lambda}d\lambda}{13-8}$	(23)

The total infrared emissivity is

$\varepsilon_{IR}=\frac{\int\varepsilon_{\lambda}B_{\lambda}(T)d\lambda}{\int B_{\lambda}(T)d\lambda}$	(24)

where *B_λ_(T)* is the blackbody radiation spectrum.

**Note S3: Calculation of net cooling power**

The sample is regarded as an interface layer within the complex sample-environment system. Four primary heat transfer pathways are considered, including radiation, conduction, convection, and evaporation. When exposed to a clear sky, the net cooling power (*P_net_*) can be calculated by the following equation:

$P_{net}=P_{rad}-P_{atm}-P_{sun}-P_{non-rad}-P_{load}$	(28)

where, *P_load_* represents the additional external heat load applied to the sample, such as heat input from an underlying heating source or device operation. This term accounts for practical working conditions where the sample is subjected to extra thermal input beyond environmental heat exchange. *P_rad_* is the radiation power of the sample and can be calculated by:

$P_{rad}=2\pi\int_{0}^{\frac{\pi}{2}} d\theta sin \theta cos \theta\int_{2.5}^{25} d\lambda I_{BB}\left( \lambda, T \right) \varepsilon\left( \lambda, \theta\right)$	(29)

where, *I_BB_ (λ, T)* is the spectral radiance of black body by law of Planck at *T*. $\varepsilon\left( \lambda, \theta\right)$ is the emissivity of the sample and can be replaced by the absorptivity according to Kirchhoff's radiation law:

$\varepsilon(\lambda)=A(\lambda)=1-T(\lambda)-R(\lambda)$	(30)

*P_atm_* is the atmospheric radiation power and can be defined as:

$P_{atm}=2\pi\int_{0}^{\frac{\pi}{2}} d\theta sin \theta cos \theta\int_{2.5}^{25} d\lambda I_{BB}\left( \lambda, T_{amb} \right) \varepsilon\left( \lambda, \theta\right) \varepsilon_{atm}\left( \lambda, \theta\right)$	(31)

where, *ε_atm_* is the spectral and angular emissivity of the atmospheric environment, which can be calculated as:

$\varepsilon_{\alpha tm}=1-t\left( \lambda\right)^{\frac{1}{\cos\theta}}$	(32)

where, *t(λ)* is the atmospheric transmittance in the zenith direction.

*P_sun_* is the solar radiation power. The calculation formula is as follows:

$P_{sun}=2\pi\int_{0}^{2.5} d\lambda I_{solar}\left( \lambda, \theta\right) \varepsilon\left( \lambda, 0 \right)$	(33)

where, *I_solar_* is the solar radiation intensity and *ε(λ, 0)* is the solar absorptivity of the sample at 0°.

*P_non-rad_* is the non-radiative heat transfer power due to convection and conduction and can be calculated as:

$P_{non-rad}=h_{c}\cdot\left( T_{atm}-T \right)$	(34)

where, *T_amb_* and *T* refer to the ambient temperature and the temperature of the sample, respectively. *h_c_* is the non-radiative heat transfer coefficient, usually 0-12 W m^-2^ K^-1^.

**Figure S1.** GPC traces of SEBS.

**Figure S2.** Schematic illustration of the preparation process of SEDO.

**Figure S3.** Interdomain spacing and phase diameter of SEDO with different DOPO loadings.

**Figure S4.** XRD patterns of DOPO and SEDO with different DOPO loadings.

**Figure S5.** DSC curve of DOPO.

**Figure S6.** (a) Infrared reflectivity and (b) transmissivity spectra of SEDO (PS content 25%) with different DOPO loadings.

**Figure S7.** Infrared absorption spectra of SEDO with different DOPO loadings.

**Figure S8.** Simulated infrared reflectivity transmissivity, and emissivity spectra of SEDO with different DOPO loadings.

**Figure S9.** Infrared reflectivity and transmissivity spectra of SEDO with PS contents of (a, b) 14% and (c, d) 10% at different DOPO loadings.

**Figure S10.** ^1^H NMR spectra of SEBS with different PS contents.

**Figure S11.** Room temperature SAXS patterns of SEDO with different PS contents (a) before and (b) after annealing.

**Figure S12.** SEM images of SEDO at solvent-to-nonsolvent ratios of (a) 0, (b) 1:9, and (c) 1:6.

**Figure S13.** Spectra of solar absorptivity, transmissivity and reflectivity of SEDO with different porosities.

**Figure S14.** Effect of film thickness on the solar reflectivity of SEDO.

**Figure S15.** Solar reflectivity spectra of SEBS at different solvent-to-nonsolvent ratios.

**Figure S16.** (a) Daytime and (b) nighttime net cooling power of SEDO.

**Figure S17.** Wind speed and humidity on (a) October 28, 2025 (clear sky) and (b) October 29, 2025 (cloudy).

**Figure S18.** (a) Solar reflectivity and (b) infrared emissivity spectra of the TSD, TC, and bare heater.

**Figure S19.** Comparison of the thermal-load performance of SEDO with different PS contents and DOPO loadings under varying external heat inputs. (a) Equilibrium temperatures of SEDO(PS10), SEDO(PS14), and SEDO(PS25) containing 5 vol% DOPO. (b) Equilibrium temperatures of SEDO(PS25) containing different DOPO loadings.

**Figure S20.** Schematic diagram of a sample building model (4 m × 6 m × 2.8 m) for EnergyPlus simulation.

**Figure S21.** Effect of SEDO film thickness on the cooling energy-saving ratio across 23 representative cities simulated using EnergyPlus.

**Figure S22.** (a) Stress-strain curves during 50 consecutive loading-unloading cycles at a strain of 100%. (b) Photographs demonstrating the reversible stretching behavior of the SEDO composite under 100% strain. (c) Solar reflectivity spectra and (d) Infrared emissivity spectra of the SEDO composite before and after cyclic stretching.

**Figure S23.** (a) TG and (b) DTG curves of SEDO with different DOPO loadings.

**Figure S24.** (a) Heat release rate, (b) total heat release, (c) CO_2_ yield, (d) smoke production rate, and (e) total smoke production of SEBS and SEDO.

**Figure S25.** (a) Solar reflectivity and (b) infrared emissivity spectra of SEDO before and after 720 h of outdoor exposure.

**Figure S26**. (a) Solar reflectivity and (b) infrared emissivity spectra of SEDO before and after 168 h of UV irradiation.

**Figure S27.** SAXS patterns of SEDO at different heating temperatures.

**Figure S28.** XRD patterns of SEDO at different heating temperatures.

Table S1. Comparison of the optical, mechanical, fabrication, and flame-retardant characteristics of SEDO with representative radiative cooling materials.

| **Material** | **Filler**  **Loading**  **(vol%)** | ***R*_solar_ (%)** | $\text{ε}$**_AW_**  **(%)** | **Elongation at break (%)** | **Toughness (MJ m^-3^)** | **Fabrication method** | **Flame-**  **retardant performance** |
| --- | --- | --- | --- | --- | --- | --- | --- |
| SA/BN-Ca^2+[1]^ | ~72 | 89.7 | 84.5 | ~2.4 | ~1.5 | Pressure-assisted dehydration + ionic crosslinking | / |
| CaO-B_2_O_3_-SiO_2_ glass/Al_2_O_3_^[2]^ | ~50 | ~98 | ~93 | ~0.15 | ~3.2 | Tape casting + hot pressing + sintering | Nonflammable |
| Cellulose-PVA/  SiO_2_-*h*-BN^[3]^ | ~47 | 98.1 | 93.2 | ~3.8 | ~1.1 | Vacuum-assisted self-assembly + hot pressing | / |
| CA/PA-Ca(II)^[4]^ | ~45 | 96.5 | 98.1 | ~44.3 | ~9.3 | Blade coating | PHRR reduction: 92% |
| CNF/SiO_2_^[5]^ | ~23 | 95.6 | 95.3 | / | / | Directional freezing + freeze-drying | PHRR reduction: 53.1% |
| PVDF-HFP-AgNWs-PVB/Si_3_N_4_^[6]^ | ~8 | 92.8 | 83.8 | ~32 | ~0.5 | Electrospinning + CO_2_ laser engraving | / |
| PDMS/ZrO_2_^[7]^ | ~4.5 | 97.3 | 96.9 | ~500 | ~15.3 | Direct ink writing | No ignition after 10 s flame exposure |
| SEBS/SiO_2_^[8]^ | ~4 | ~96 | ~97 | ~660 | ~45 | Solution-based phase segregation | / |
| PMMA/PENA^[9]^ | 0 | 95.4 | 93.4 | ~60 | ~54.9 | Gradient nanolayer coextrusion + biaxial stretching + thermal lamination | / |
| **SEBS/DOPO**  **(This work)** | **~5** | **97.2** | **95.5** | **~1447.3** | **~51.2** | **Solution-based phase segregation** | **PHRR reduction: 33.2%** |

**References**

[1] L. Wang, J. Wei, X. Wu, Z. Wang, Y. Liu, J. Tang, Y. Jiang, H. Xie, W. Wang, H. Wang, H. Ping, Z. Fu, *ACS Nano* **2026**, 10.1021/acsnano.6c04344.

[2] Z. Yang, H. Lu, Q. Li, X. Guo, Q. Li, H. Zhou, B. Wang, Y. Qi, H. C. Chen, M. Yang, T. Tian, H. Yang, J. Ding, H. Pang, *Adv. Mater.* **2025**, 38, 12289.

[3] X. Lin, Z. Li, J. Li, S. He, Y. Wu, *ACS Nano* **2026**, 20, 10919.

[4] P. Du, K. Zhang, X. Li, J. Zhang, M. Song, X. Dai, J. Muhammad, F. Ge, Q. Li, Z. Cai, *Mater. Today* **2026**, 93.

[5] X. Yin, N. Feng, C. Liu, H. Li, J. Li, S. Yang, X. Zhuang, B. Cheng, *Adv. Mater.* **2026**, 10.1002/adma.74505.

[6] B. Gu, S. Feng, Q. Zhang, R. Lu, G. Xiahou, S. Huang, G. Li, H. Tang, W. Zhong, D. Zhao, *Nat. Commun.* **2026**, 17.

[7] K. Zhou, S. Tang, P. Karua, F. Wu, S. Hong, D. Patel, G. M. Reeser, D. M. Cropek, P. V. Braun, L. Cai, *Nat. Commun.* **2025**, 17, 1072.

[8] L. Xiong, C. Chen, K. Tian, X. Zhang, M. Wen, C. Guo, M. Cheng, Q. Li, Q. Fu, H. Deng, *Adv. Mater.* **2025**, 37, 2504150.

[9] G. Yu, H. Wang, W. Hong, Z. Wang, Y. Xiong, H. Wu, J. Shen, J. Wang, H. Zhao, S. Guo, C. Li, *Adv. Mater.* **2025**, 38, 18322.
